# Impact of different cooking methods on polycyclic aromatic hydrocarbons in rabbit meat

**DOI:** 10.1002/fsn3.2284

**Published:** 2021-05-03

**Authors:** Rabia Siddique, Ameer Fawad Zahoor, Hamad Ahmad, Faisal Maqbool Zahid, Emad Karrar

**Affiliations:** ^1^ Department of Chemistry Government College University Faisalabad Pakistan; ^2^ Department of Chemistry University of Management and Technology Lahore Lahore Pakistan; ^3^ Department of Statistics Government College University Faisalabad Pakistan; ^4^ Department of Food Engineering and Technology Faculty of Engineering and Technology University of Gezira Wad Medani Sudan

**Keywords:** cooking methods, GC‐MS, PAHs, rabbit meat

## Abstract

The influence of a variety of cooking methods (poaching, boiling, grilling (charcoal or gas)), frying (pan, deep frying, and stir frying) with a variety of oils (vegetable oil, extra virgin olive oil, sesame oil, extra light olive oil, and sunflower oil), microwaving, and oven roasting on polycyclic aromatic hydrocarbons (PAHs) formation in rabbit meat samples was investigated. Meat samples (including three replicates) were prepared without additives or spices. PAHs extraction was carried out by saponification method with potassium hydroxide in methanol which was followed by a silica gel column technique and the samples were quantified by using gas chromatography with mass spectrometry (GC‐MS). PAHs standards, fluorene, naphthalene, anthracene, phenanthrene, pyrene, acenaphthalene, fluoranthene, and benzopyrene, were used for this study. The other PAHs except fluorene were not observed (detection limit‐0.009 µg/g) in all the samples. Among traditional processing techniques, higher PAH contents were observed as a result of frying. Frying with vegetable oil produced higher fluorene content (0.06–0.13 µg/g) in the deep‐fried sample, although sesame oil is the best oil which produces lowest PAH contents in fried samples. Among all the processing techniques, lower fluorene (0.01–0.02 µg/g) content was noticed in poaching. Benzo(a)pyrene was not observed in all the investigated samples which is viewed as a reliable strategy of the cooking process for human consumption. After processing, the cooking loss was determined and oven roasting and grilling exhibited greater moisture loss.

## Graphical abstract

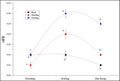

## 1 INTRODUCTION

Rabbit meat muscle food has valuable nutritional characteristics, such as high protein content (21.2 g/100 g), low content of cholesterol (56.4 mg/100 g), and fat (9.2 g/100 g) (Nistor et al., 2013). Due to its dietetic and nutritious behavior, the consumption of rabbit meat has increased around 2.8% from 2007 to 2017. In 2017, the meat consumed approximately 1.5 million tons in all over the world (Pla et al., 2004).

An anthropologist studied that consumption of meat is pivotal for the evolutionary heritage of humans. Humans began to process foods by using fire around 1.8 million years ago (Ferraro et al., 2013). The cooking of meat provides delicious food with excellent taste, good texture, and better digestibility. However, nutritive characteristics of meat are influenced by cooking treatments and can produce deleterious substances, such as polycyclic aromatic hydrocarbons (PAHs) (Sobral et al., 2018). PAHs are lipophilic compounds formed when meat is processed by using high thermal methods (Viegas et al., 2012) such as in food preparation methods (smoking and drying) and processing (roasting, frying, grilling, and microwaving) (Ishizaki et al., 2010). PAHs are generated when the processing of meat over a hot surface produces juices and fat that drip on the surface and producing smoke. PAH‐containing smoke adheres to the meat surface and food becomes contaminated (Farhadian et al., 2010).

PAHs are known as mutagens and carcinogens; they alter the DNA structure and may cause cancer (Chen & Chen, 2003). The United States Environmental Protection Agency (USEPA) has listed PAHs as priority pollutants. Benzo[a]pyrene (B[a]P) has been selected as a marker for the occurrence of PAHs in foodstuffs (No, 1881).

This study aimed to focus on the influence of diverse cooking procedures, like poaching, boiling, grilling (charcoal or gas), microwaving, frying (pan, deep, and stir frying), and oven roasting on the generation of PAHs contaminants. Different parameters (temperature, distance between rabbit meat and heating source, as well as processing time) were set for this study. The second purpose was to check the effect of various oils including vegetable oil (a combination of sunflower oil, soybean oil, and canola oil), sesame oil, sunflower oil, extra virgin olive oil, and extra light olive oil.

## 2 MATERIALS AND METHODS

### 2.1 Chemicals

Fluoranthene, phenanthrene, naphthalene, and silica gel (70‐230 mesh ASTM) were obtained from Merck. Fluorene and pyrene from Hopkin Williams LTD, whereas acenaphthalene, benzopyrene, and anthracene were bought from BDH. HPLC grade solvents, like *n*‐hexane, dichloromethane, and acetonitrile, were purchased from Samchun. Potassium hydroxide was bought from Scharlau while methanol and anhydrous sodium sulfate from Daejung. Rabbit meat was bought from the local market of Faisalabad, Pakistan.

### 2.2 Sample preparation

For sampling, thirty rabbits (each weighing around 350 g) were purchased from a supermarket in Faisalabad, Pakistan. The meat samples of rabbit (foreleg, hind leg, and back) were separately cleaned with tap water and then dipped into acetic acid (2% solution) for 10 min and removed layers of fat and bones from meat (Abou‐Arab et al., 2014). After dehydrating, the rabbit meat samples were homogenized for 2 min with Waring food grinder. Every part of the rabbit sample was deliberated equally, placed in transparent nylon bags, and frozen at −20°C (Siddique et al., 2021).

**FIGURE 1:**
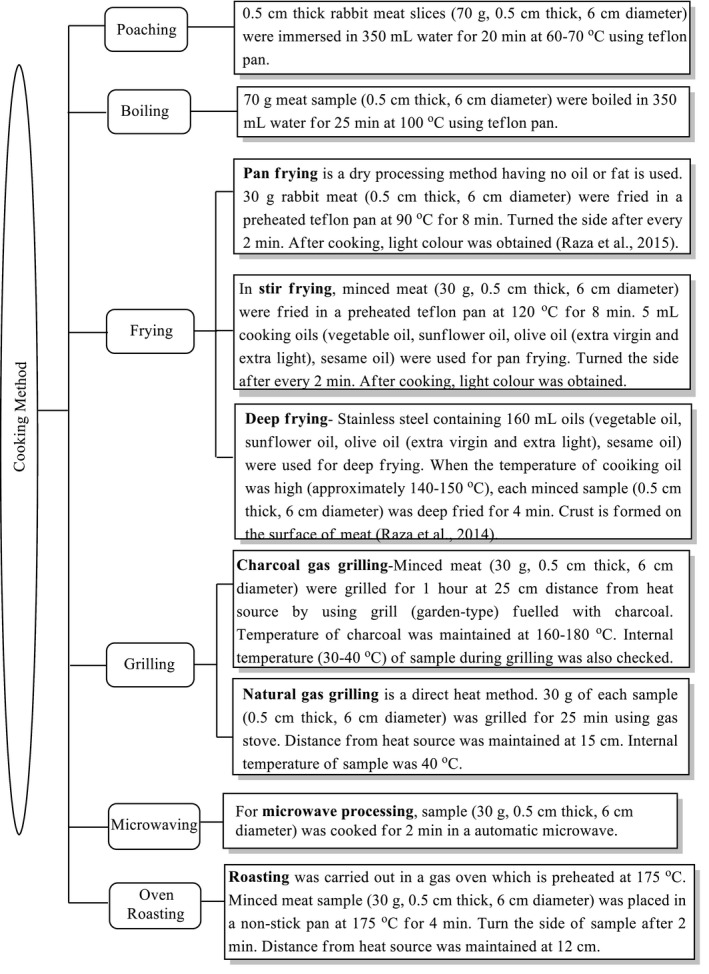
Flow chart diagram of cooking procedures

In this study, processing methods including poaching, boiling, frying (pan, deep, and stir frying) with a variety of oils, grilling (charcoal grilling and natural gas grilling), microwaving, and oven roasting were applied for samples (three portions for each sample) preparation (Sobral et al., 2018). The list of cooking methods is represented in Figure 1. No spices or condiments were added to the processing treatments. After processing, the samples were cooled and deliberated the cooking mass. Then, the samples were homogenized in a Waring food grinder (Milford, MA, USA). Every sample (including three replicates) was placed in transparent nylon bags and frozen at −20°C (Duedahl‐Olesen et al., 2015).

### 2.3 Sample extraction and clean‐up procedure

**FIGURE 2:**
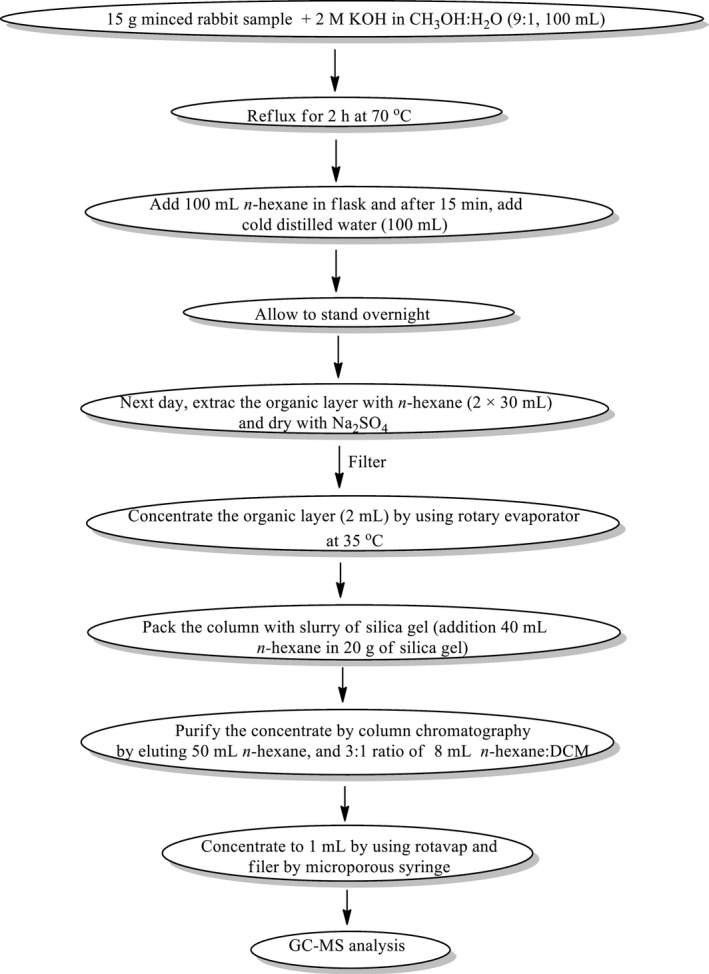
A schematic diagram for clean‐up of samples

The extraction and clean‐up methodology which is utilized for the separation of PAHs from samples is a slight variation of a procedure used by Chung et al., (2011). A schematic diagram of the sample extraction procedure for PAH determination is shown in Figure 2.

### 2.4 Standard preparation

Stock solutions of PAHs standards (acenaphthalene, phenanthrene, fluorene, naphthalene, anthracene, pyrene, fluoranthene, and benzopyrene) were prepared. 100 µg/ml stock solutions was prepared by adding each standard (100 µg) in acetonitrile (1 ml). To prepare working standard solution (20 µg/ml) of every standard, each stock standard solution (1 ml) was diluted with 4 ml acetonitrile. These prepared standard solutions were utilized to evaluate the calibration curve (Siddique et al., 2020).

### 2.5 Analytical method

Polycyclic aromatic hydrocarbons compounds in samples of rabbit meat (108 samples) were quantified with the GC‐MS technique by Kao et al., (2012) with some modification. An Agilent 7890 B gas chromatograph with 5977 mass spectrometers and 30 m × 0.25 mm ID × 0.25 µm film thickness, DB‐5MS capillary column was used for the separation of PAHs. Split less injection (1 µl sample) with helium as a carrier gas (flow rate 1 ml/min) was used. The gas chromatograph oven was operated at 80°C for 1 min, raised to 260°C for 1 min at 25°C/min, then 300°C for 6.3 min at 10°C/min. The other conditions for the separation of PAHs are shown below: injector temperature 290°C and detector temperature: 150°C for ion source and 230°C for quadrupole. Run time of chromatogram optimized at 25 min and the minimum detection for each sample was 0.009 µg/g. The quantification of PAHs was carried out using the calibration method.

### 2.6 Statistical analysis

Analysis of variance (One and Two way ANOVA) was used for relating various cooking kinds and meat samples. The analysis was done using the statistical software Minitab 13.2.

## 3 RESULTS AND DISCUSSION

### 3.1 Cooking loss

The loss of meat samples during processing is depicted in Table 1. During thermal treatment, loss of meat samples does not only depend on various cooking treatments, the surface of cooking, time, and temperature but also based on the quality of meat, including the content of fat, protein, and water, as well as pH and size of meat (Alfaia et al., 2010). Processing losses seem to rise directly with the processing time and the internal temperature attained by meat products, with oven roasting and grilling having greater moisture loss and higher intramuscular content of lipids (Alfaia et al., 2010).

**TABLE 1:** Cooking loss in various methodologies

Cooking treatment	Cooking loss
Poaching	32%
Boiling	30%
Frying	43%
Grilling	49%
Microwaving	34%
Oven roasting	50%

Samples of meat were prepared according to the various cooking methods listed in Figure 1. It is very difficult to relate our results with the previous studies; to our knowledge, it is the first research on PAHs analysis in rabbit meat by using a variety of processing treatments. PAHs have two main classes: Low PAHs (naphthalene, fluorene, fluoranthene, phenanthrene, anthracene, acenaphthalene, and pyrene) and heavy PAHs (benzo[b]pyrene). PAHs are formed when meat is processed at 150–300°C (high temperature) (Viegas et al., 2012).

### 3.2 Poaching, and boiling

Poaching is a simple moist heat cooking method by submerging meat in the water at low temperatures (70–80°C). Due to low temperature, meat is gradually soft and moist without losing its moisture level. Poaching produced low fluorene content (0.01 µg/g) in all the poached samples.

**FIGURE 3:**
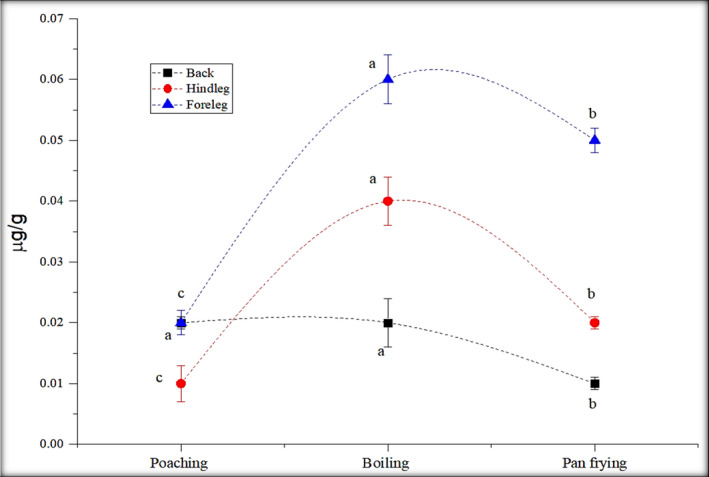
Impact of boiling, poaching, and pan‐frying methods on fluorene concentration (µg/g)

Boiling is a simple technique used for cooking a variety of foods. Solyakov and Skog (2002) reported that boiling of poultry items does not produce a large number of carcinogenic compounds, because in this cooking technique, low temperature (<100°C) is used. In this study, the only fluorene was detected in the range of 0.02–0.06 µg/g. The other PAHs were produced below the detection limit (0.009 µg/g) in all the boiled samples. Low PAHs formed in poached samples rather than boiled samples because low temperature and less time were used in poaching (Figure 3).

### 3.3 Frying

Frying is a rapid cooking methodology that provides a delicious taste. Frying is classified into three classes: (a) pan frying having no oil or fat, (b) stir frying with minimum oil or fat, (c) deep frying having maximum oil or fat. Pan frying generated low content of PAHs. Benzo[a]pyrene content in raw edible oils is given in Table 2. Among all these edible oils, vegetable oil exhibited a higher concentration of benzo[a]pyrene (0.0005 µg/g) followed by sunflower oil (0.0004 µg/g). Olive oil (extra virgin and extra light) showed the lowest benzo[a]pyrene (0.0001 µg/g).

**TABLE 2:** Benzo[a]pyrene concentration in raw edible oils

Edible oils	Benzo[a]pyrene (µg/g)
Vegetable oil	0.0005
Sunflower oil	0.0004
Sesame oil	0.0002
Olive oil (extra virgin)	0.0001
Olive oil (extra light)	0.0001

### 3.4 Pan‐fried

As shown in Figure 3, fluorene was only detected in all the parts (hind leg, back, foreleg) of rabbit meat in the range of 0.05–0.07 µg/g. Pan‐fried samples exhibited a low level of contaminated particles than stir frying (Figure 3) (Raza et al., 2015). Pan‐frying process used no oil or fat at the base of samples, so that transfer of heat into the inner portion of muscles is less as compared to stir and deep frying with using oil as a medium and exhibited a smaller concentration of fluorene formed in rabbit meat processed through this technique.

### 3.5 Stir‐fried and deep‐fried

**FIGURE 4:**
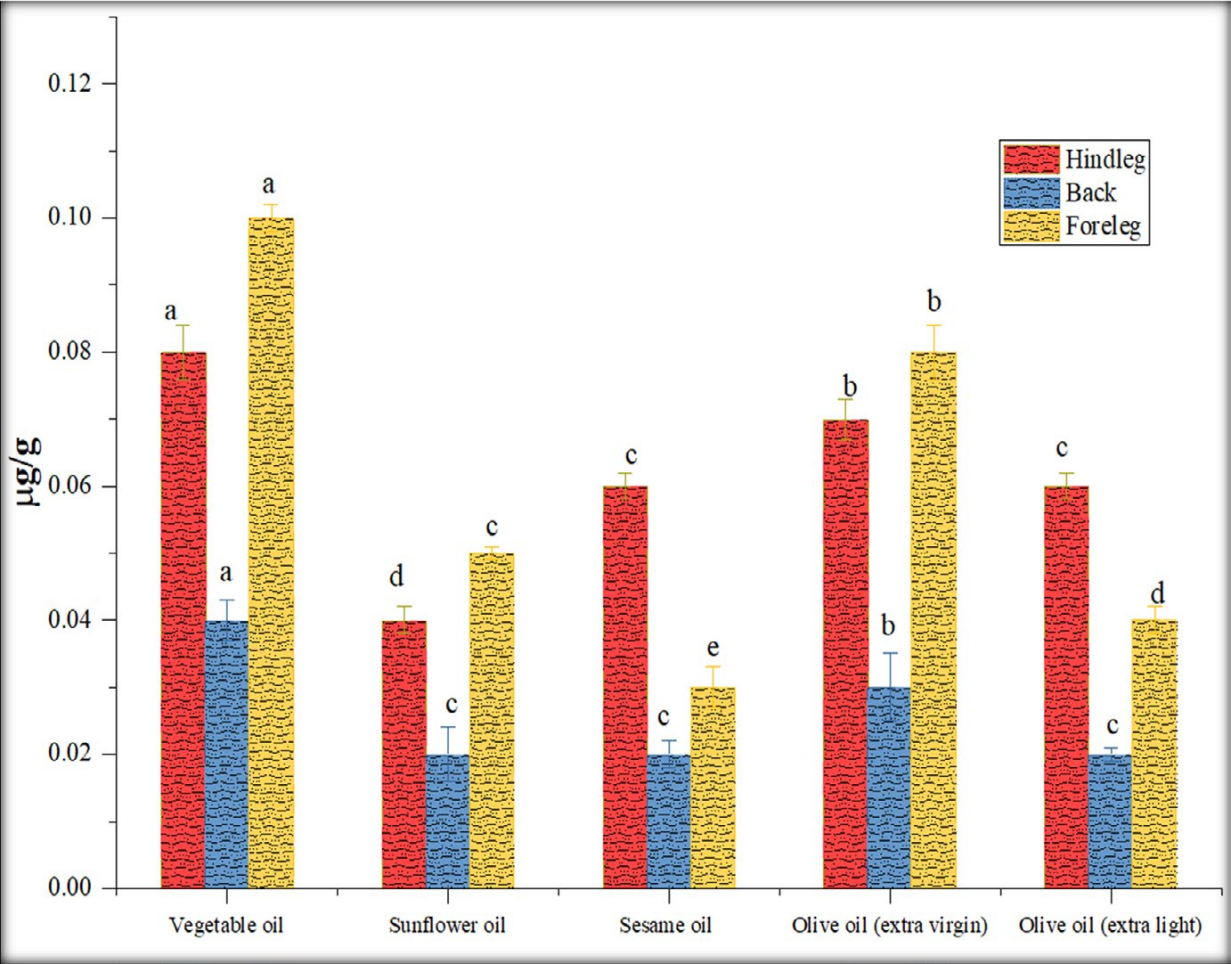
Effect of stir frying with different oils on fluorene (µg/g)

The low PAHs and benzo[a]pyrene contents emitted from stir‐frying and deep‐frying processes are shown in Figure 4. PAH emissions produced from diverse cooking procedures were noticed differently, and PAH levels in deep‐frying procedures were significantly greater than those formed *via* frying process, across oil temperature as well as volume investigated. As presented in Figure 1, the average temperature of the oil was reached at 140–150°C during the deep‐frying process, which is higher than the temperature (120°C) used during the other frying cooking methodologies. In the study of Abdullahi et al., (2013) and Yao et al., (2015), deep‐frying cooking procedure produced more PAH concentration due to the usage of greater temperature and volume of oil. Hence, oil volume and its temperature significantly affect PAH concentration.

**FIGURE 5:**
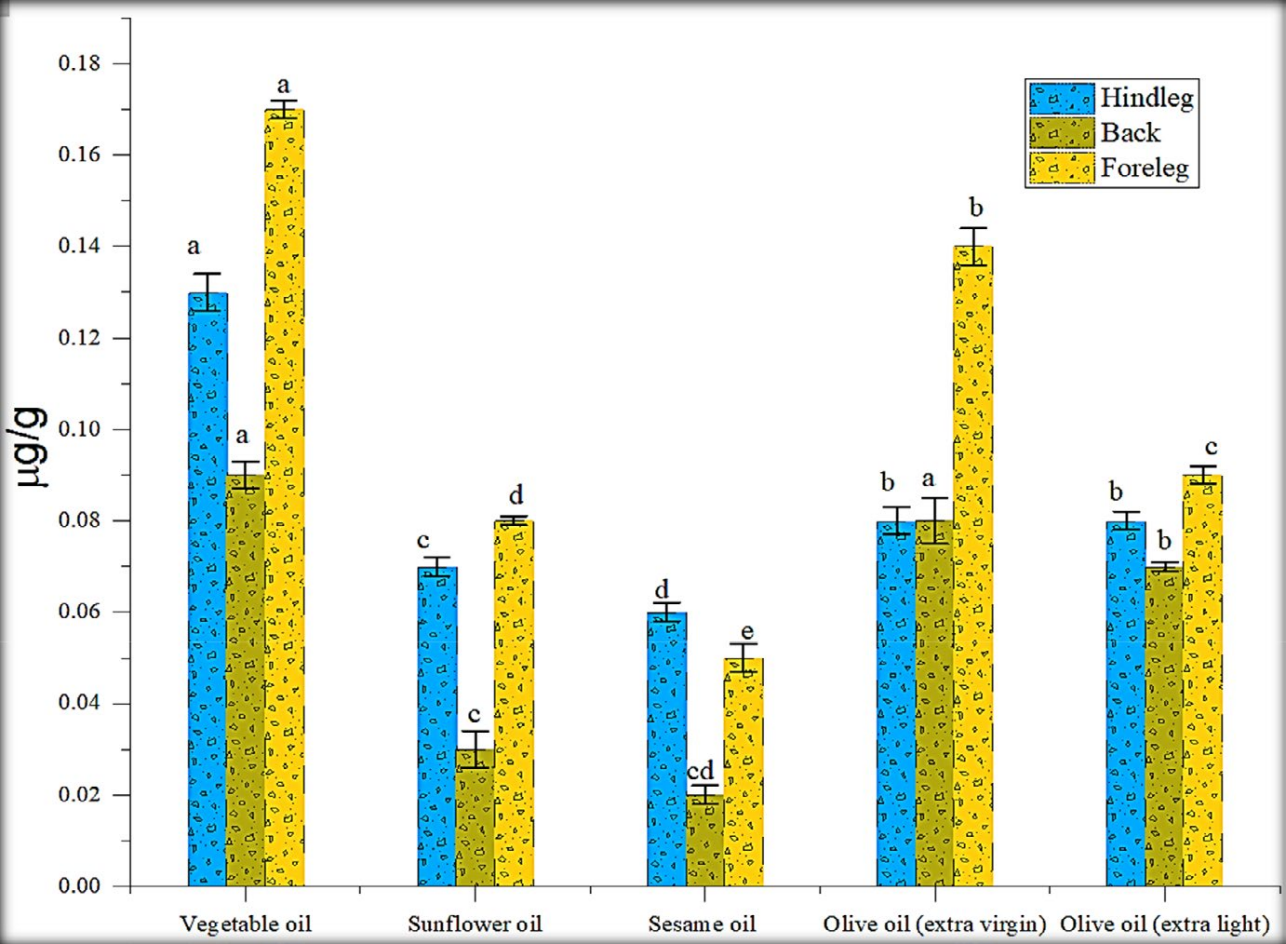
Effect of deep frying with different oils on fluorene (µg/g)

The fluorene range in hind leg portion was 0.03–0.13 µg/g, 0.02–0.09 µg/g in back and 0.05–0.17 µg/g in foreleg. The PAHs contents in rabbit meat were significantly lower than Janoszka et al., (2004) study. Samples were fried with vegetable oil contained the highest fluorene content as compared to other oils. Stir frying with vegetable oil generated 0.08 µg/g fluorene content in the hind leg, 0.04 µg/g in the back, and 0.10 µg/g in the foreleg. Whereas, deep frying with vegetable oil generated 0.13 µg/g fluorene content in the hind leg, 0.09 µg/g in the back, and 0.17 µg/g in the foreleg. Processing with vegetable oil produced the greatest PAH level, followed by processing with extra virgin olive oil, extra light olive oil, sunflower oil, and sesame oil. Deep‐fried foreleg rabbit meat produced the highest content of PAH, followed by hind leg deep frying, and back deep‐frying process. By comparing sesame oil with extra virgin olive oil, olive oil produced high PAH concentration in meat (Hao et al., 2016). The fluorene content was also produced at a high concentration (0.08 µg/g) in the foreleg portion by using sunflower oil; sunflower oil possessed a high PAH level (Fromberg et al., 2007) (Figure 5).

The deep‐frying process utilized more fat than other cooking techniques. Lipids were absorbed into the inner division of meat samples, so it reduced the protein degradation, and less extreme variations occurred. But foreleg portion of rabbit meat contains larger fat and protein content than the other portions, both of which are accountable for the PAH generation. Protein molecules are degraded to form amino acids, which then react with sugars compounds that are already in the muscle and produce precursors in the generation of contamination. Lipid dilutes these starting materials and transfers extra heat into the meat fiber, which promote them to act together at higher speed and produce PAH at a higher amount (Raza et al., 2015).

Among all the PAHs, benzo[a]pyrene is the most toxic PAH, and it is considered as an indicator of total PAH in the environment (Tiwari et al., 2013). Abdullahi et al., (2013) described that greater amount of heavy PAHs was produced in the deep‐frying process. Moreover, See et al., (2006) explained that lower and heavy PAH compounds were produced by providing the low‐ and high‐temperature processing methodologies.

In our study, benzo[a]pyrene was not detected in all the samples. By comparison of all the results, it can be confirmed that parameters (temperature and volume of oil) set for frying conditions are more suitable and produced safer food for human consumption.

### 3.6 Grilling

**FIGURE 6:**
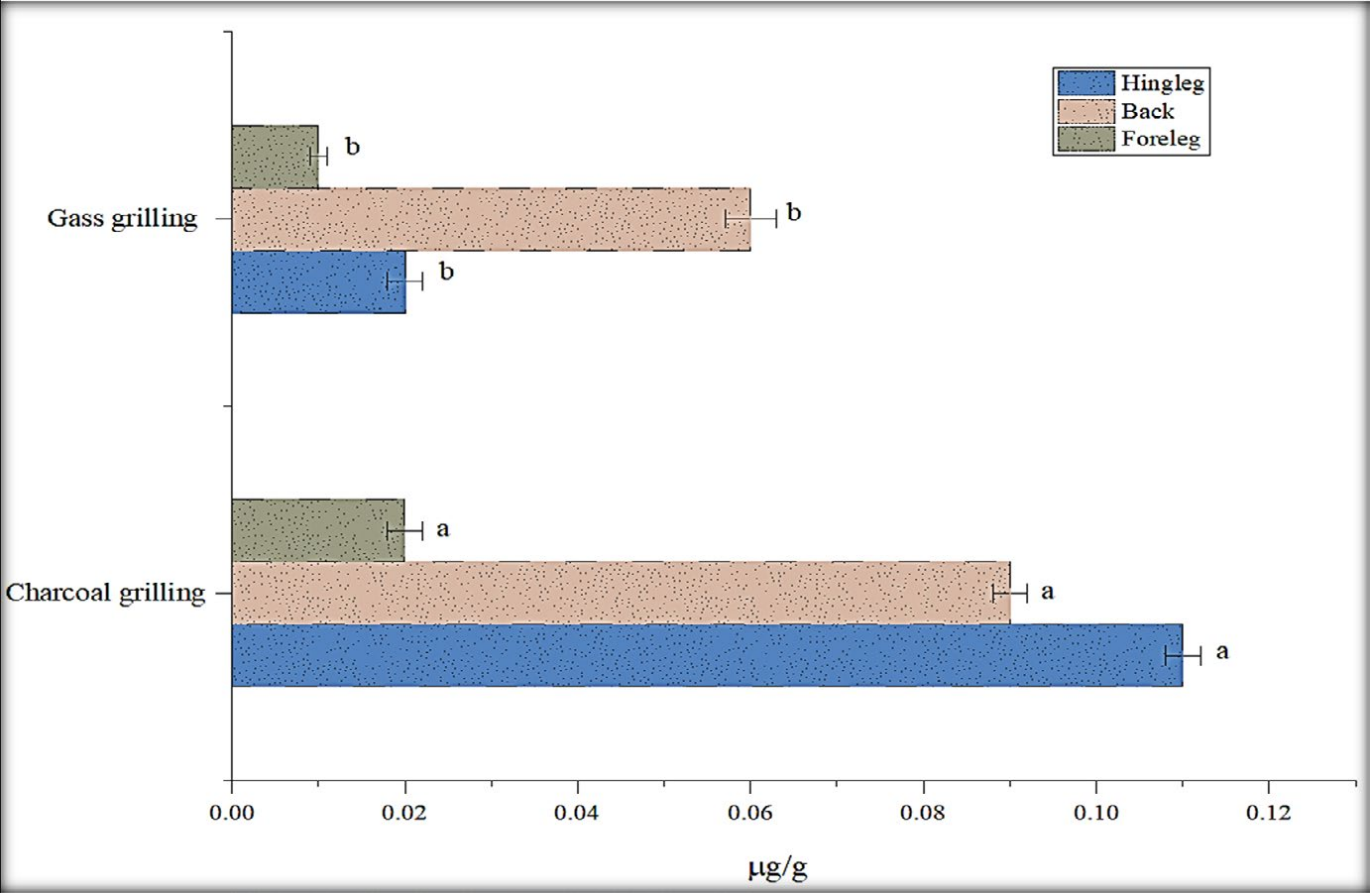
Effect of charcoal and natural gas grilling on fluorene content (µg/g)

Grilling was seen to be the most dominant processing method in the generation of these mutagens. It is a quick meal preparation technique that uses a direct heat source. Meat is cooked directly over gas grills or charcoal at high temperatures. Temperature and the distance between the heat source and meat are the other major factors to PAH formation (Chung et al., 2011). The generation of PAHs is greatly dependent on the type of grilling; gas grilling as well as charcoal grilling (Gorji et al., 2016; Viegas et al., 2012). In this study, charcoal grilling produced significantly higher fluorene content (0.02–0.11 µg/g) in all the parts (Figure 6). Grilling with charcoal leads to higher PAH generation which resulted from incomplete burning of charcoal as compared to gas grilling (Gorji et al., 2016). In charcoal grilling, the dripping of fat molecules on fire generates higher PAH values (Farhadian et al., 2010). According to previous literature, wood charcoal grilling presented PAH levels at a high range (Sobral et al., 2018). Grilling with natural gas produced only fluorene concentration (0.01–0.06 µg/g) in rabbit meat.

By observing all the parameters, in our research work, the distance was increased 15 cm in gas grilling and 25 cm at 160–180°C in charcoal grilling. Benzopyrene, a toxic PAH, was not detected in all the grilled samples. Maintain a low temperature (<200°C) and a high distance between a heat source and the food can be responsible for the reduction of PAH levels. In conclusion, the safety of grilled food from carcinogenic contaminants can be controlled by maintaining the temperature and the distance from the heat source.

### 3.7 Microwaving and oven roasting

**FIGURE 7:**
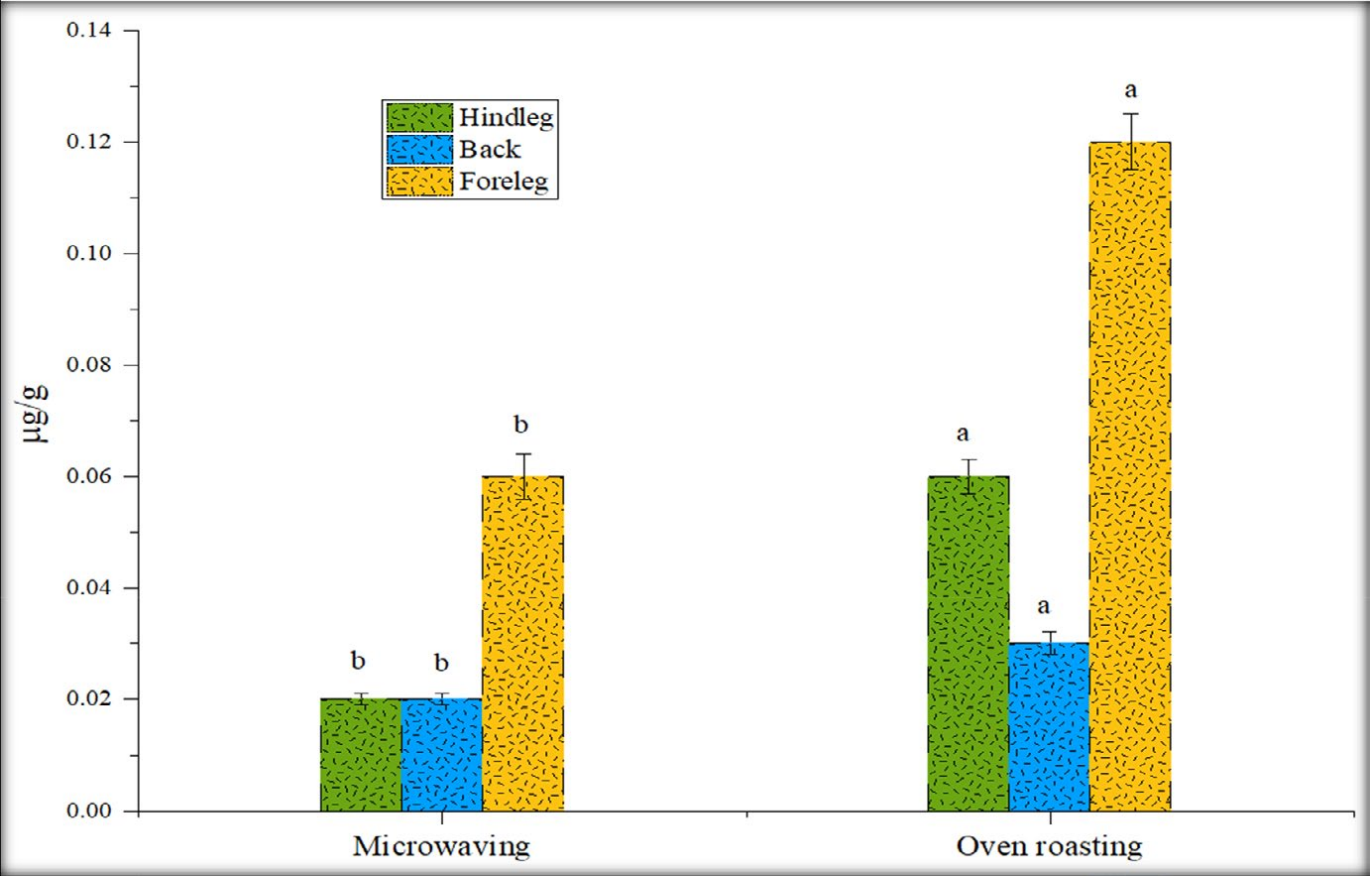
Effect of microwaving and oven roasting on fluorene concentration (µg/g)

For microwave processing, cooking of rabbit meat in a microwave was carried out in an automatic mode for 2 min. All PAHs except fluorene were not detected in all the microwaving samples. Fluorene content was significantly higher in the foreleg (0.06 µg/g) as compared to back (0.02 µg/g) and hind leg (0.02 µg/g) samples (Figure 7).

Roasting using an oven is a common cooking technique that is used for enhancing the flavor. In this cooking, neither fat nor oil is used for oven roasting. Oven roasting resulted in the generation of the only fluorene among the quantified PAHs in rabbit meat processed in the oven. The fluorene concentration was showed to be 0.12 µg/g in the foreleg, 0.03 µg/g in the back, and 0.06 µg/g in hind leg samples. By comparing the GC‐MS results of microwaving and oven roasting, microwaved rabbit meat samples showed higher fluorene content than oven roasting (Figure 7).

## 4 CONCLUSION

It was difficult to compare our results with the previous studies because there is no such type of literature in rabbit meat by using a variety of processing treatments. The effect of diversified cooking techniques, a variety of oils, and temperature are important factors for controlling PAHs concentration in meat. Deep frying is rapid processing that involves meat is submerged in a huge amount of oil, which produced the significantly highest impact on PAHs in rabbit meat. Vegetable oil is not suitable oil for deep frying; it produced high PAH contents. Hence, sesame oil produced lower PAHs concentration. Among all the cooking procedures, poaching is the best processing methodology that exhibits lower PAHs levels. Control of processing time, less temperature, and more distance can overcome the PAH values. All the parameters set for various cooking techniques were the greatest step to control the PAH range. Benzo[a]pyrene (carcinogenic compound) was not detected in all the samples.

## CONFLICT OF INTEREST

Authors declared no conflict of interest exists.
